# Genomic Organization of H2Av Containing Nucleosomes in *Drosophila* Heterochromatin

**DOI:** 10.1371/journal.pone.0020511

**Published:** 2011-06-27

**Authors:** Zhenhai Zhang, B. Franklin Pugh

**Affiliations:** Center for Comparative Genomics and Bioinformatics, Center for Eukaryotic Gene Regulation, Department of Biochemistry and Molecular Biology, The Pennsylvania State University, University Park, Pennsylvania, United States of America; National Institutes of Health, United States of America

## Abstract

H2Av is a versatile histone variant that plays both positive and negative roles in transcription, DNA repair, and chromatin structure in *Drosophila*. H2Av, and its broader homolog H2A.Z, tend to be enriched toward 5′ ends of genes, and exist in both euchromatin and heterochromatin. Its organization around euchromatin genes and other features have been described in many eukaryotic model organisms. However, less is known about H2Av nucleosome organization in heterochromatin. Here we report the properties and organization of individual H2Av nucleosomes around genes and transposable elements located in *Drosophila* heterochromatic regions. We compare the similarity and differences with that found in euchromatic regions. Our analyses suggest that nucleosomes are intrinsically positioned on inverted repeats of DNA transposable elements such as those related to the “1360” element, but are not intrinsically positioned on retrotransposon-related elements.

## Introduction

In *Drosophila*, as in many other eukaryotes, chromosomes are packaged into euchromatin and heterochromatin [1], [2], [3]. Most genes reside in euchromatin, and are thought to exist in a more open state than those found in compact heterochromatin. Genes that are ectopically placed within heterochromatin tend to be repressed [4], [5], [6], [7], which reflects a possible spreading of the heterochromatin structure into such genes. Heterochromatic regions are gene poor. However, many resident genes are expressed, and typically rely on the heterochromatin environment for proper expression [8].

Heterochromatin contains many repeat sequences [9], and this includes a variety of transposable elements. DNA transposons use the host cell's replication machinery to replicate itself through a DNA intermediate, which involves terminal inverted repeats or TIRs [10]. Retrotransposons multiply via an RNA intermediate, and are divided into two groups: LTR retrotransposons that contain ‘long terminal repeats’ at their end, and non-LTR retrotransposons (LINE and SINE elements) that lack LTRs [11]. A number of studies suggest that transposable elements are involved in chromosome organization and function [12], [13], [14], [15], [16], [17], epigenetic regulation of specific genes [18], [19], [20], and human diseases [21], [22], [23]. Knowledge of their nucleosome organization may provide some insight into their contribution to genome integrity and epigenetic regulation.

After DNA replication, many regions in the genome replace their H2A and H3 with the histone variants H2A.Z and H3.3 [24], [25], [26], [27], [28], and this provides additional functionality to those nucleosomes. Typically, these replacement histones reside at active genes, and therefore may have special roles in regulating gene expression. However, this is not strictly the case in that H2A.Z can accumulate in an undirected manner at transcriptionally quiescent regions of the genome, including heterochromatin [29]. While H2A.Z is found in both euchromatin and heterochromatin [29], [30], [31], [32], [33], it is not clear whether it serves the same purpose in both locations, as it has been implicated in both activation and repression [34], [35], [36]. In euchromatin, H2A.Z is enriched at the 5′ ends of genes [37], [38], [39], but its precise positioning and function is less well defined in heterochromatin. Nevertheless, H2A.Z might protect euchromatin from the ectopic spread of silent heterochromatin in nearby regions [28].

In *Saccharomyces*, H2A.Z is incorporated into the −1 and +1 nucleosomes of euchromatic genes [40]. These positions flank a nucleosome-free promoter region (NFR) [41]. In metazoans, H2A.Z is relatively depleted upstream of the core promoter region, and is concentrated at the +1 position with a decreasing gradient of occupancy at nucleosome positions downstream of +1 [42], [43]. However, chromatin that is isolated without *in vivo* crosslinking and in the presence of low ionic stringency appears to accumulate H2A.Z in promoter regions [44], indicating that promoter regions may be accessible for H2A.Z assembly. In *Drosophila*, H2A.Z is referred to as H2Av [45]. H2Av incorporates functions of both H2A.Z and H2A.X that are encoded separately in other systems. In this paper we will use “H2Av” when referring specifically to the *Drosophila* protein, and “H2A.Z” when used more generally.

Inasmuch as H2A.Z tends to be more easily evicted from chromatin [38], it may ease the entry of a transcribing polymerase into a gene [46], compared to canonical histones. Nonetheless, the +1 nucleosome is positioned to potentially control RNA polymerase II entry into a gene [42], [47], [48], [49], [50]. For example, repositioning of the +1 nucleosome has been implicated in control of cell cycle genes [51]. Moreover, RNA polymerase II is paused at the 5′ end of genes [52], [53], just upstream of the +1 nucleosome [42]. Inasmuch as nucleosome positioning can dictate the accessibility of chromosomal elements, knowing their precise position should shed light onto how genes, DNA replication, DNA repair, and transposition are regulated.

Previously, we reported a high-resolution euchromatin-wide map of H2Av nucleosome positions using MNase ChIP-seq technology in which nucleosome occupancy was covalently trapped at its *in vivo* location [42], and this provided some insight into how chromatin is organized around cis-regulatory elements and genomic features. Here we report on the heterochromatin portion of the dataset and describe the sequence and organizational properties of H2Av around annotated (FlyBase r5.14) heterochromatic genes and transposable elements in comparison to their euchromatin counterparts. Given the diverse functions of H2Av in all aspects of chromosome biology, the ill-defined nature of heterochromatin, and the limitations to which extracted H2Av mono-nucleosomes can be quantitatively measured, we limit our analysis here to a qualitative comparison between mapped H2Av locations and surrounding annotated features. Our analysis is intended to provide a resource describing H2Av nucleosome organization and properties in heterochromatic regions (as compared to euchromatic regions) and its implications, rather than providing a deep mechanistic understanding of H2Av function.

## Results and Discussion

### Heterochromatic and euchromatic H2Av nucleosomes are very similar

**Figure 1:**
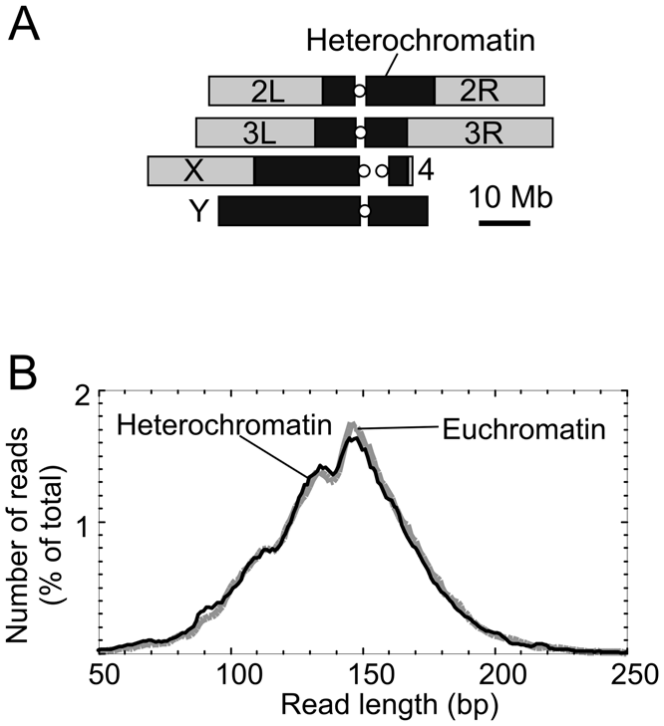
Length distribution of H2Av nucleosomal DNA in heterochromatic and euchromatic regions. **A**, Schematic of heterochromatic (black) and euchromatic (gray) regions of the *Drosophila* genome. **B**, Read length distribution of reads aligned to euchromatic and heterochromatic genomic regions, respectively. The Y-axis represents percentile of total mapped reads in euchromatin and heterochromatin, separately.

In characterizing heterochromatic H2Av nucleosomes, we first examined their instrinsic properties, then examined their distribution around genomic features. *Drosophila* heterochromatin predominates in pericentric regions, and encompasses most or all of the Y chromosome [3] (**Figure 1A**). Approximately 33% of the female *Drosophila* genome is heterochromatic, compared ∼46% in males [3]. *Drosophila* heterochromatin consists of tandemly repeated short sequences (satellite DNAs), middle repetitive elements (e.g. transposable elements), and some single-copy sequences. Previously, we had covalently crosslinked nucleosomes to their native *in vivo* locations using formaldehyde, then isolated the chromatin and digested it to nucleosome core particles using micrococcal nuclease (MNase) [42]. In accord with standard protocol for genome-wide mapping of nucleosomes, >80% of the chromatin was reduced to mono-nucleosomes using high concentrations of MNase, and the vast majority of nucleosomes were rendered soluble, indicating that the chromatin was being uniformly sampled. Importantly, the nucleosomes were immunoprecipitated with H2Av antibodies under stringent conditions that removed any non-covalently crosslinked nucleosomes. By ensuring that the H2Av nucleosomes were crosslinked *in vivo*, we avoided complications associated with potential repositioning and/or redeposition of H2Av nucleosomes during the *in vitro* work up.

Inasmuch as the entire length of the nucleosomal DNA was sequenced, the maps simultaneously demarcated both nucleosome borders in a single read. This provided twice the information as short-read sequencing, and thus provided high accuracy, which we have demonstrated to have a median error of ∼4 bp [40]. Attributing the nucleosome dyad position to the midpoint between the two sequenced ends also diminished (but did not eliminate) effects of MNase cleavage bias. We determined whether the length of DNA protected by the histone octamer was the same in heterochromatic and euchromatic regions. As shown in **Figure 1B**, the distribution of sizes of heterochromatic H2Av nucleosomes was nearly identical to that of its euchromatic counterpart, with both peaking at approximately 147 bp. The consistency of length distributions between both types of nucleosomes indicates that under the conditions employed other factors are not likely to be bound to bulk heterochromatic H2Av nucleosomes in a way that alters their protection pattern. In both types of nucleosomes, shoulders were evident at ∼132 bp and 112 bp, representing nucleosomal DNA that is ∼15 and ∼35 bp shorter than the most frequent length, respectively. This likely represents some “breathing” of the DNA at the nucleosome borders, thereby making them more nuclease accessible.

**Figure 2:**
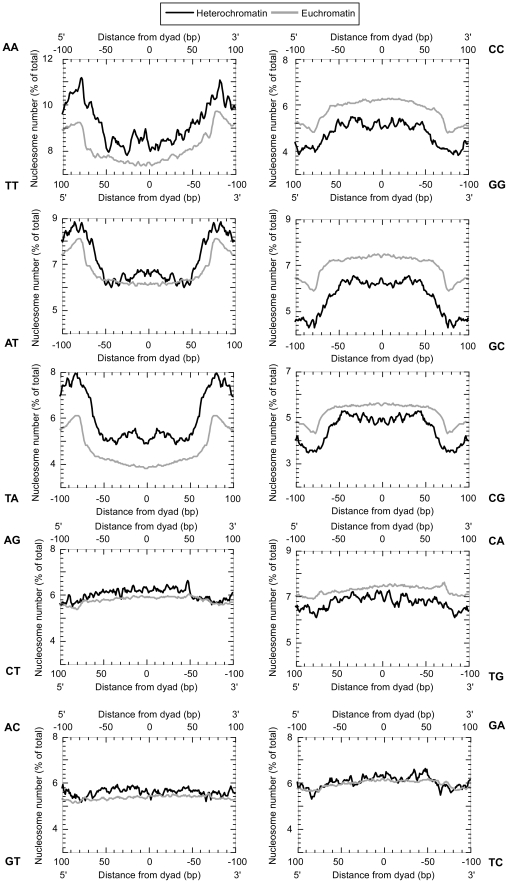
Dinucleotide frequency distribution. The dinucleotide count was calculated for both the forward and reverse strands, reading from the 5′ to 3′ direction. The frequency distribution of one dinucleotide (e.g. 5′-AC-3′) plotted at the top from left to right is equal to its complement (e.g. 5′-GT-3′) reported from right to left at the bottom. Self-complementary dinucleotides are reported on only one axis.

Patterns of dinucleotides have been associated with nucleosome positioning [40], [42], [54], [55], [56], [57], [58], . We therefore compared the dinucleotide pattern previously determined for euchromatic H2Av nucleosomes with those found in heterochromatin. As shown in **Figure 2**, the dinucleotide distributions across nucleosomes were essentially identical between euchromatic and heterochromatic H2Av nucleosomes. However, heterochromatic regions displayed an overall higher content of AA/TT/TA dinucleotides, which are well-defined components of nucleosome positioning sequences. In both heterochromatic and euchromatic chromatin, nucleosomal DNA tended to be more G/C-rich and A/T-deficient than linkers (regions >73 bp from the dyad). Thus, any sequence-based positioning by the underlying nucleosomal DNA is likely to employ the same basic rules in heterochromatin as it does in euchromatin, but may be more pervasive in heterochromatic regions.

### H2Av nucleosomes tend to be more restricted at heterochromatic vs euchromatic genes

**Figure 3:**
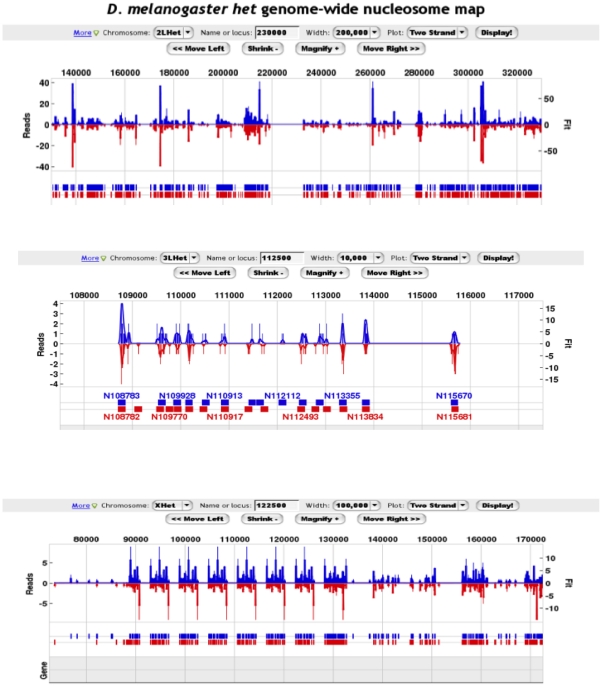
Examples of detected H2Av locations in heterochromatic regions. Three panels are shown, exemplifying different heterochromatic regions and at different zoom levels. The blue graph represents the number of forward strand reads mapped to each coordinate (and shifted 3′ to a higher coordinate by 73 bp to reflect the nucleosome midpoint). The red bar graph, scaled in the opposite direction, for comparison, represents the number of reverse strand reads mapped to each coordinate (shifted 3′ to a lower coordinate by 73 bp). Nucleosomes were predicted through GeneTrack using an exclusion zone of 147 bp and sigma of 20 bp [67], [68]. The blue and red hash marks indicate nucleosome midpoint positions. Two examples are shown.

As shown in **Figure 3**, nucleosomal sequences obtained independently from the forward and reverse strands were co-incident at predicting nucleosome midpoints, which provides independent validation of the H2Av nucleosome positions. This two-strand verification indicated that most locations lacking H2Av were not likely due to low coverage. However, a number of caveats preclude quantitative interpretation of the coverage. First, nucleosomes from region to region may differ in their extractability from the nucleus. Second, in a population, nucleosomes may differ in the extent to which H2A.Z is incorporated. Third, nucleosomal sequences may differ in their efficiency of amplification by PCR. The latter however is controlled to some extent by the generation of independent maps on each strand, since they are derived from the same population of nucleosomes for a given position. An unambiguous deconvolution of these multiple contributions was not possible, and this limits what can be gleaned from occupancy data. Nucleosome positioning, however, is less senstive to these parameters and thus is the primary focus of this study. Nonetheless, we made occupancy comparisons that were averaged across large genomic regions or averaged across a large number of similar positions (e.g. +1 nucleosome of all mRNA genes in a particular chromatin class), which to some extent alleviated fluctuations at individual nucleosome locations.

**Figure 4:**
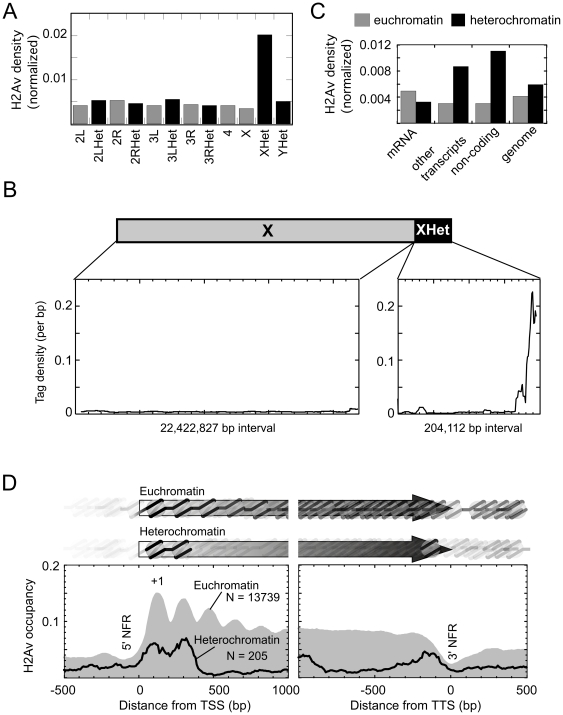
H2Av nucleosome density on chromosomes and organization at mRNA genes. **A**, Weighted H2Av read counts were normalized to the amount of sequence present in each chromosomal region, and plotted. **B**, Nucleosomal DNA tag counts at each coordinates were plotted for both euchromatin and heterochromatin regions on the X chromosome. The height of blue bar indicates the total normalized tag count at each coordinate. Regions for euchromatin and heterochromatin is scaled differently along the X-axis. The Y-axis is on same scale. **C**, Euchromatin and heterochromatin genomes were divided into three categories: mRNA, other transcripts (tRNA, miRNA, transposons, etc), and non-coding regions. H2Av densities were calculated as total tag numbers divided by total base pair in all features in that particular category. **D**, Shown are a composite plots of the H2Av nucleosome occupancy distribution relative to TSSs (transcription start site, left panel) and TTSs (transcript termination site or polyA site, right panel) for the set of annotated heterochromatic and euchromatic genes. The number of genes included is indicated by N. Nucleosomal DNA read counts were weighted, binned, then normalized to the number of regions present in each bin, and plotted as a smoothed distribution. In order to represent a “pure” pattern, we removed those regions that were <300 bp from an adjacent TSS or TTS. Light gray-filled plot represents the distribution of reads in euchromatin. The black trace represents the distribution in heterochromatin.

We examined the density of H2Av at each chromosome, separated by euchromatic and heterochromatic regions. H2Av densities were similar at all chromosomes regardless of chromatin type (**Figure 4A**), with the apparent exception of heterochromatin located on the X chromosome, which had four times the density of H2Av. The high levels of H2Av in the heterochromatic portion of the X chromosome were largely at one end (**Figure 4B**). rDNA repeats are not enriched in this region, and thus the increased level of H2Av cannot be accounted for by unannotated copies of rDNA.

We compared the density of H2Av around features located in heterochromatic vs euchromatic regions of the genome (**Figure 4C**). While much of the *Drosophila* genome including mRNA genes contained similar average densities of H2Av in heterochromatin vs euchromatin, H2Av was enriched nearly three fold in heterochromatic non-mRNA genes, which includes transposable elements. The functional significance of this enrichment is not clear, but might be related to a recently described phenomenon whereby H2Av tends to accumulate in under-transcribed regions [29].

We mapped the distribution of H2Av nucleosomal sequences around transcription start sites (TSSs) of genes residing in heterochromatin, and compared it with existing maps in euchromatic genes (**Figure 4D**, left panel). Genes located in heterochromatic regions had very low levels of H2Av at their −1 nucleosome, as seen previously for euchromatic genes [42]. The +1 nucleosome was located at +135 bp relative to the TSS, as also seen at euchromatic genes. This would place the nucleosome border downstream of the TSS, and in position to potentially impede transcription elongation by RNA polymerase II but not initiation. The inter-nucleosomal spacing between +1 and +2 nucleosomes was exactly the same as that in euchromatin (∼175 bp). Thus the mechanism that establishes the canonical H2Av nucleosome positioning and spacing at euchromatic genes could apply to heterochromatic genes as well. Interestingly, downstream of the +2 nucleosome position, H2Av levels dropped precipitously on mRNA genes located in heterochromatic regions, rather than the gradual tapering seen for euchromatic genes. This was not due to heterochromatic genes being shorter. This differential suggests that the nucleosome composition around genes located in heterochromatic regions differ from those located in euchromatic regions.

When the 3′ ends of genes were examined (**Figure 4D**, right panel), a pattern similar to but flipped in comparison to the 5′ end was observed, in that an H2Av nucleosome peak was evident on the genic side of a 3′ NFR residing at the end of the genes. This enrichment of H2Av at the 3′ end of heterochromatic genes may reflect a special function of the terminal nucleosome.

### H2Av nucleosomes mark both ends of DNA-TIRE elements

**Figure 5:**
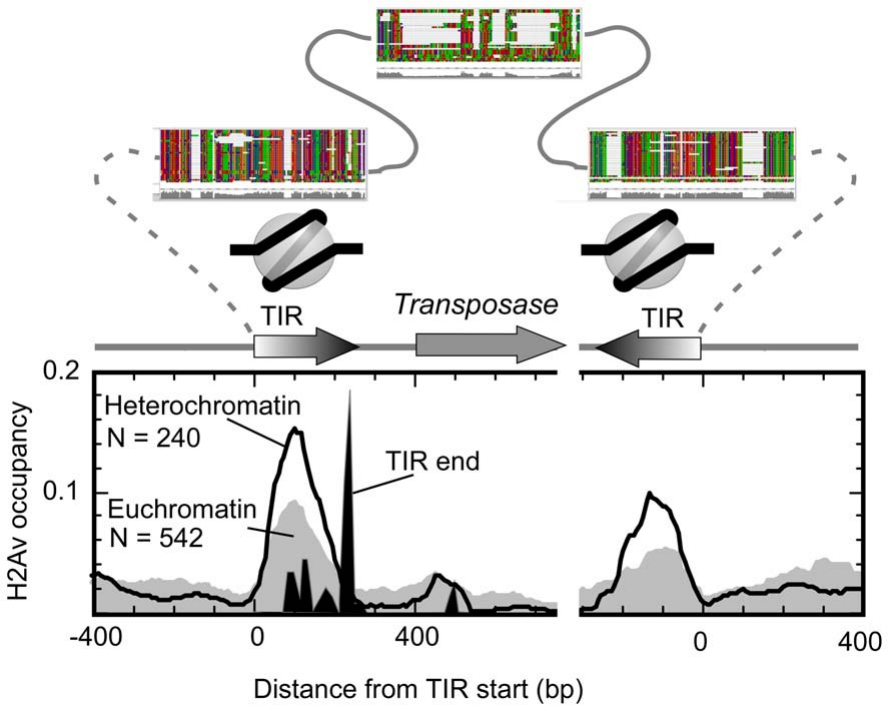
H2Av nucleosome organization at TIRE transposable elements. A composite distribution of H2Av nucleosomal sequence tags is shown around the start (left panel) and end (right panel) of DNA TIR elements. Euchromatic elements are shown in the gray-filled plot and heterochromatic elements are indicated by the black trace. The relative location of TIR ends is shown as a black filled plot. The structure of the TIREs, drawn to approximate scale is shown above the plot. Multiple sequence alignments of TIREs is shown above the schematic, illustrating the proposed location of an H2Av nucleosome. Many TIREs are degenerate, lacking the internal homology regions to the transposon, and lacking a matching downstream inverted repeat.

We examined the distribution of H2Av around DNA-TIR elements, of which the majority are composed of the “1360” element (**Figure 5**). These transposons replicate through DNA intermediates. We observed a similar pattern in both euchromatic and heterochromatic TIREs, with a single major positioned H2Av nucleosome centered 100 bp downstream of the element start. This would place the start of the TIR on the outside edge of the nucleosome, in a place readily accessible by the transposase. Interestingly, the distance of the H2Av nucleosome from the TIR start is roughly similar to that of the TSS at the 5′ end of genes. Because TIREs exist in many locations with presumably distinct chromatin environments, these results suggest that the DNA sequences within the terminal inverted repeats of TIREs define the position of the resident nucleosome. The presence of well positioned H2Av on the “1360” TIRs fits well with observations that such elements produce small RNAs involved in RNAi-directed heterochromatic silencing [66], in that H2Av is linked to sites of transcription.

### Lack of a consensus H2Av organization around retrotransposons

**Figure 6:**
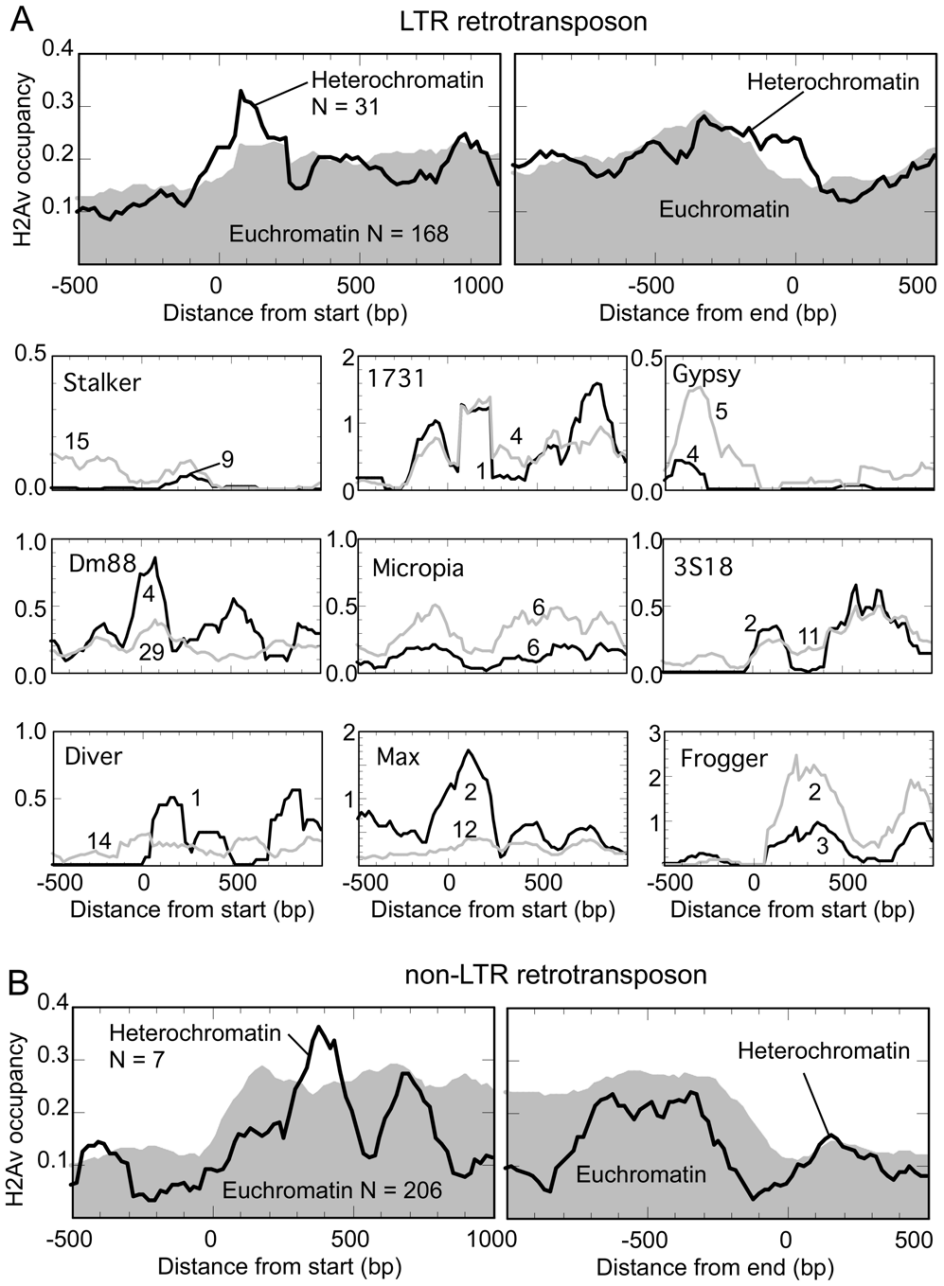
H2Av nucleosome organization flanking LTR and nonLTR retrotransposons. **A**, Composite H2Av nucleosome occupancy distribution around the start (top left panel) or end (top right panel) of LTR elements. Euchromatic regions are plotted as gray fill, and heterochromatic regions as a black trace. N indicates the number of elements represented. The lower set of nine plots show the distribution of H2Av around the start of specific classes of LTRs. Numbers on the graph indicate the number of cases found, and are positioned closest to the trace it reflects. **B**, Composite H2Av nucleosome occupancy distribution around the start (left) or end (right) of non-LTR elements.

**Figure 6A, B** shows the H2Av nucleosome landscapes surrounding LTR and non-LTR retrotransposons, respectively. A robust consensus organization was not evident for bulk LTRs, indicating that each may either lack positioned nucleosomes or lack a consensus on positioning relative to the element start site. Some H2Av enrichment was observed just downstream of the LTR start. When broken out by various LTR classes in **Fig. 6A**, distinct patterns were observed. For some classes, euchromatic and heterochromatic patterns were essentially the same, but for others the patterns looked specific towards one type of chromatin. Because the number of elements was small in each case, we cannot rule statistical fluctuation. The lack of robust patterning suggests that the surrounding environment in which retrotransposons are located within may influence the chromatin structure and thus the expression of the retrotransposon.

### Conclusions

H2Av nucleosomes are distributed throughout euchromatic and heterochromatic regions, as established in a number of studies. Our findings indicate that the basic organization of the H2Av nucleosome is indistinguishable in these two regions. Moreover, H2Av nucleosomes generally adopt essentially the same positions relative to specific classes of genomic features (e.g., genes and transposons) in both types of environments, indicating that such features may dictate the positioning of resident nucleosomes. Retrotransposons appear to be more of an exception where positions relative to their start or end points are not intrinsic to the elements. Perhaps the local chromatin environment may influence the position of nucleosomes on these elements. Strikingly, whether it be genes, transposons, or replication origins, H2Av (and H2A.Z) nucleosomes seem to mark their boundaries, perhaps facilitating access of the relevant regulatory machinery.

## Methods

### Data source

Raw data was a by-product of the genome-wide MNase ChIP-seq study described in reference [42]. Bulk downloads or specific queries of nucleosomes positions can be accessed from http://drosophila.atlas.bx.psu.edu.

### H2Av nucleosomal reads weight calculation

Individual nucleosomal DNA reads were mapped to *Drosophila* heterochromatin and euchromatin genomes (r5.14) respectively via BLAST. Only reads with >90% alignments and >90% identity were retained and hit numbers for all reads were recorded. Each uniquely aligned read was weighted as one. The weight of multiple aligned reads were calculated as one divided by total hit number. Thus, if a reads was mapped to five different locations, it was weighted as 0.2 (1/5).

### Feature coordinates

Transcriptional start sites (TSSs) and termination sites (TTS) for mRNAs were downloaded from FlyBase (r5.14). Transposon sequences were also downloaded from FlyBase. For DNA terminal inverted repeat elements (DNA TIREs) and long terminal repeat retrotransposons (LTR retrotransposons), each sequence was divided into two halves. Then these two halves were aligned to eliminate repeats at both ends, if any. Only sequences longer than 200 bp after removal of the end repeat were used for further analysis. To determine the start and end position of each element, transposons were aligned to *Drosophila melanogaster* genome (euchromatin and heterochromatin respectively), and resultant start and end sites were used for further analysis. For DNA-TIRE element, overlapping alignment regions for identical element were merged together. For LTR and non-LTR retrotransposons, only alignments that are longer than 800 bp were retained for further analysis.

### Summary of additional data files

The following additional data are available with the online version of this paper. Additional data file 1 (Data S1) reports the heterochromatin coordinates of each nucleosome called by Gene Track. Additional data file 2 (Data S2) reports the euchromatic genomic features used in this study. Additional data file 3 (Data S3) reports the heterochromatic genomic features used in this study.

## Supporting Information

Data S1 Reports the heterochromatin coordinates of each nucleosome called by Gene Track.
(TXT)

Data S2 Reports the euchromatic genomic features used in this study.
(TXT)

Data S3 Reports the heterochromatic genomic features used in this study.
(TXT)
